# NRF2/ARE mediated antioxidant response to glaucoma: role of glia and retinal ganglion cells

**DOI:** 10.1186/s40478-023-01663-1

**Published:** 2023-10-24

**Authors:** Sarah Naguib, Jon R. Backstrom, Elisabeth Artis, Purnima Ghose, Amy Stahl, Rachael Hardin, Ameer A. Haider, John Ang, David J. Calkins, Tonia S. Rex

**Affiliations:** 1 Neuroscience Program, Vanderbilt University, Nashville, TN USA; 2 Vanderbilt University Medical Center, Vanderbilt Eye Institute, Nashville, TN USA

**Keywords:** glaucoma, Nrf2, Oxidative stress, Retinal ganglion cell, Optic nerve, Antioxidant, Glia

## Abstract

Glaucoma, the second leading cause of irreversible blindness worldwide, is associated with age and sensitivity to intraocular pressure (IOP). We have shown that elevated IOP causes an early increase in levels of reactive oxygen species (ROS) in the microbead occlusion mouse model. We also detected an endogenous antioxidant response mediated by Nuclear factor erythroid 2-Related Factor 2 (NRF2), a transcription factor that binds to the antioxidant response element (ARE) and increases transcription of antioxidant genes. Our previous studies show that inhibiting this pathway results in earlier and greater glaucoma pathology. In this study, we sought to determine if this endogenous antioxidant response is driven by the retinal ganglion cells (RGCs) or glial cells. We used Nrf2^fl/fl^ mice and cell-type specific adeno-associated viruses (AAVs) expressing Cre to alter Nrf2 levels in either the RGCs or glial cells. Then, we quantified the endogenous antioxidant response, visual function and optic nerve histology after IOP elevation. We found that knock-down of Nrf2 in either cell type blunts the antioxidant response and results in earlier pathology and vision loss. Further, we show that delivery of Nrf2 to the RGCs is sufficient to provide neuroprotection. In summary, both the RGCs and glial cells contribute to the antioxidant response, but treatment of the RGCs alone with increased Nrf2 is sufficient to delay onset of vision loss and axon degeneration in this induced model of glaucoma.

**Supplementary Information:**

The online version contains supplementary material available at 10.1186/s40478-023-01663-1.

## Introduction

Glaucoma is a neurodegenerative disease characterized by the progressive degeneration and eventual death of retinal ganglion cells (RGCs), the neurons necessary for the processing and transmitting of visual information from the retina to the brain (for review see: [1–4]). The disease results in irreversible blindness and affects almost 80 million people worldwide, with that number projected to increase to 111 million by 2040 [5]. The major risk factors for developing glaucoma are age, genetics, and sensitivity to intraocular pressure (IOP). Current treatments for glaucoma aim to decrease IOP. However, poor patient compliance with IOP-lowering drops combined with lack of responsiveness to IOP-lowering drugs and surgeries leads to permanent vision loss in many patients. Thus, further research aimed at protecting RGCs, independent of IOP, is an approach that needs to be explored [1–4].

While the underlying molecular cause of RGC degeneration and death in glaucoma is complex, it is clear that accumulation of reactive oxygen species (ROS) plays a role [6–8]. We previously showed that the antioxidant NRF2/ARE pathway is activated after elevation of IOP. Nuclear factor erythroid 2-Related Factor 2 (Nrf2), when activated, binds to the Antioxidant Response Element (ARE), an enhancer element that is upstream of many antioxidant genes. Nrf2 affects the degree and onset of neurodegeneration in many models, including models of RGC injury and glaucoma [9–15]. Under homeostatic conditions, Nrf2 remains in the cytoplasm, where it is sequestered by its repressor protein, Kelch-like ECH associated protein 1 (Keap1) and is constantly targeted for degradation via ubiquitination. When Keap1 is oxidized, it undergoes a conformational change that releases Nrf2, allowing it to accumulate and translocate into the nucleus of the cell where it can bind to the ARE, and activate antioxidant gene transcription. Nrf2 can also be released from Keap1 upon phosphorylation from upstream signaling molecules, resulting also in nuclear translocation [9, 16–18]. We also found that Nrf2 is phosphorylated by the PI3K/Akt pathway at 2 weeks post-IOP elevation in the microbead occlusion model of glaucoma [8]. Further, inhibiting this pathway prevented increases in ARE-driven transcripts, showing that the retina endogenously responds to increased IOP via activation of the NRF2-ARE pathway through phosphorylation by PI3K/Akt. We have also shown that NRF2-ARE activation in glaucoma is context dependent—in the presence of erythropoietin, we found that the NRF2-ARE pathway is activated through phosphorylation by MAPK instead [19].

In this study we sought to determine the cell-type specificity of the NRF2/ARE pathway activation in the mouse microbead occlusion model of glaucoma. We used Nrf2^fl/fl^ mice and RGC or glial-directed AAVs to deliver Cre to each cell type separately. We then quantified functional and molecular changes in both groups after IOP elevation. Finally, we demonstrate the efficacy of AAV2/2.Nrf2 gene therapy in the context of a global Nrf2 knock-out or overexpression in wild-type mice following microbead occlusion.

## Materials and methods

### AAV construct development and AAV production

pAAV.CMV.Nrf2 was purchased from Addgene (Watertown, MA; plasmid #67,636) and packaged into AAV2/2 at SignaGen (Fredrick, MD). pAAV.CMV.eGFP was purchased from Addgene (plasmid #67,634) and packaged into AAV2/2 at SignaGen. The AAV.ARE.tdTomato reporter virus was constructed as previously described [19]. The construct was tested in ARPE-19 cells prior to use in vivo (Supplemental Fig. 1). ARPE-19 cells were purchased from ATCC (Manassas, VA) and grown as previously described [20]. Cells in complete media were transfected with plasmid DNA mixed with FuGENE HD (Promega, Madison, WI; #E2311) at a ratio of 1:4 (1 mg DNA: 4 ml FuGENE). Eight-well chamber slides and 6-well plates were transfected with 200ng and 1 mg DNA per well, respectively. One day after transfection, the media was replaced with serum-free (SF) medium and incubated for 24 h. Cells were treated with SF medium containing either 5 mM sulforaphane, an NRF2 activator, (MedChemExpress, Monmouth Junction, NJ; #HY-13,755) or vehicle (0.025% DMSO) and incubated for an additional 24 h. Cells were preserved with Histochoice, washed with PBS, and coverslips mounted with ProLong Gold (Thermo Fisher). For immunoblots, 10 µg of PBS-soluble protein was analyzed per lane. Blots were probed with rabbit anti-RFP (Rockland Immunochemicals, Inc., Limerick, PA; #600-401-379) and mouse anti-HA (Cell Signaling Technologies, Danvers, MA; #2367). ARE activation, based on increased tdTomato (tdTom) fluorescence, was detected in transfected cells treated with sulforaphane, but not in those that did not receive sulforaphane nor those that were transfected with the plasmid carrying the inactive M4 ARE (Supplemental Fig. 1). Cells transfected with WT ARE and treated with either DMSO or sulforaphane produced both tdTom and ZsGreen, with more intense tdTom in the cells treated with sulforaphane (Supplemental Fig. 1). However, in the ARPE19 cells transfected with M4 ARE, there was no tdTom band (Supplemental Fig. 1).

AAVs were generated that express Cre recombinase and tdTom (see Table 1). The promoters included either the 1 kb human vimentin (Vim) promoter that was derived from Addgene plasmid #29,114 [21] or the 0.66 kb mouse gamma-synuclein (Sncg) promoter (Addgene plasmid #153,163) [22]. RGCs were targeted with the combination of Sncg promoter and AAV2/2 capsid, which has been widely used to efficiently infect RGCs after intravitreal injection. Additionally, the Sncg promoter has been characterized such that following intravitreal injection, more than 85% of the transduced RGCs also double-labeled with RBPMS [22]. While we aimed to primarily transduce astrocytes with the combination of a 1 kb Vim promoter and a modified AAV6 capsid (ShH10 with an additional Y455F mutation) [31], we have not excluded the possibility that some Müller glia were also transduced following intravitreal injection. Control AAVs contained tdTomato without Cre (see Table 1). All Cre and control AAVs were packaged in-house using triple transfection of HEK cells.

**Table 1:** Plasmids used to produce AAVs in this study

Plasmid	AAV nomenclature
pAAV.pTrx-ARE(WT)-TnSV0HA-zG	AAV2/2.ARE
pAAV.pCMV.Nrf2	AAV2/2.Nrf2
pAAV.pCMV.eGFP	AAV2/2.eGFP
pAAV.pSncg.Cre.IRES.tdTomato	AAV2/2.Sncg.Cre
PAAV.pVimentin.Cre.P2A.tdTomato	AAV2/6m.Vim.Cre
pAAV.pSncg.tdTomato	AAV2/2.Sncg.tdTom
pAAV.pVimentin.tdTomato	AAV2/6m.Vim.tdTom

### Mice

Control mice (C57Bl/6J), *Nrf2* knockout mice (B6.129 × 1-*Nfe2l2*^*tm1Ywk*^*/J*) or *Nrf2* floxed mice (C57BL/6-*Nfe2l2*^*tm1.Sred*^*/SbisJ*) (Jackson Labs, Bar Harbor, ME) were group-housed, maintained on a 12-h light-dark cycle, and provided food and water *ad libitum*. An equal distribution of 2–3 month old male and female mice were used for this project.

### Microbead occlusion model (MOM)

IOP was bilaterally elevated using the well-characterized microbead occlusion model (MOM) of glaucoma [23, 24]. We injected 2 µl of 15-µm diameter FluoSpheres polystyrene microbeads into the anterior chamber of anesthetized mice (Thermo Fisher, Waltham, MA) as previously described [23–25]. Additional mice received bilateral injections of an equivalent volume of lactated Ringer’s saline solution as controls. Briefly, 1.5 mm outer diameter/1.12 mm inner diameter filamented capillary tubes (World Precision Instruments, Sarasota, FL) were pulled using a P-97 horizontal puller (Sutter Instrument Company, Novato, CA), and the resulting needles were broken using forceps to an inner diameter of ~ 100 μm. Microbeads were loaded and injected using a microinjection pump (World Precision Instruments, Sarasota, FL). Mice were anesthetized with isoflurane and dilated using topical 1% tropicamide ophthalmic solution (Patterson Veterinary, Devens, MA), and 2 µl (~ 2,000 microbeads) were injected. The needle was maintained in the injection site for 20 s before retraction to reduce microbead efflux. Mice were given topical 0.3% tobramycin ophthalmic solution (Patterson Veterinary, Devens, MA) following injection.

### IOP measurements

IOP was measured immediately prior to microbead injection and biweekly thereafter using the Icare TonoLab rebound tonometer (Colonial Medical Supply, Franconia, NH) as previously described [23–25]. Mice were anesthetized using isoflurane, and 10 measurements were acquired from each eye within 2 min of induction of anesthesia.

### AAV injections

For experiments with an endpoint of 5 weeks post-IOP elevation, viruses were intravitreally injected 1 wk prior to MOM injections. For experiments with an endpoint of 2 wks post-IOP elevation, viruses were intravitreally injected 2 weeks prior to MOM injections. All mice used in this study were injected with 1ul of virus at a concentration of 1 × 10^9^ GC/ul.

### In vivo electrophysiology

Mice were dark adapted overnight, dilated with 1% tropicamide for 10 min and anesthetized with 20/8/0.8 mg/kg ketamine/xylaxine/urethane according to previously published methodology [8, 19]. Mice were placed on the heated surface of the ERG system to maintain body temperature. Corneal electrodes with integrated stimulators (Celeris System, Diagnosys LLC, Lowell, MA) were placed on eyes that were lubricated with GenTeal drops. Subdermal platinum needle electrodes were placed in the snout and back of the head at the location of the visual cortex. A ground electrode was placed in the back of the mouse. For VEPs, mice were exposed to 50 flashes of 1 Hz, 0.05 cd.s/m^2^ white light with a pulse frequency of 1 flash. For flash ERGs and VEPs, mice were first exposed to flashes of 1 Hz, 1 cd.s/m^2^ white light with a pulse frequency of 1 following dark adaptation. Secondly, after the mice had already been exposed to the flash ERG/VEP, we recorded the photopic negative response (PhNR) of the ERG by exposing mice to 100 continuous flashes of white light on a green background with a pulse frequency of 2. Each experimental group had 12–16 eyes.

### Dihydroethidum fluorescence

A dye that fluoresces in the presence of superoxide and, to a lesser extent, hydrogen peroxide, dihydroethidum (DHE), was utilized for these studies as previously described [31]. Mice were anesthetized with 2.5% isofluorane and intravitreally injected with 1 µl (0.5 μm) of DHE (ThermoFisher Scientific, Waltham, MA) diluted in phosphate-buffered saline (PBS) using a 30-gauge Hamilton syringe. Just prior to imaging, mice were anesthetized with ketamine/xylazine and eyes were dilated with 1% tropicamide. Thirty minutes after DHE injection, fluorescence was imaged on a Micron IV retinal imaging microscope (Phoenix Research Labs, Pleasanton, CA) using an FF02-475/50 nm excitation filter (Semrock, Inc. Rochester, NY) and ET620/60X emission filter (Chroma Technology Corp., Bellows Falls, VT). The average intensity of the fluorescence throughout the retina was quantified using ImageJ [32]. For each experimental group, 6–8 eyes were analyzed. Notably, the DHE fluorescence was measured for each group during the same imaging session.

### Tissue collection

For western blots and qPCR, retinas were collected fresh and flash-frozen from mice euthanized by anesthetic overdose and cervical dislocation. For immunohistochemistry and optic nerve histology, tissue was fresh collected and post-fixed in 4% paraformaldehyde until use at 4^o^C.

### Protein assay

Protein concentrations were determined from 10 µl of retina homogenates with the Pierce BCA Protein Assay Kit (cat#: 23,225, ThermoFisher Scientific, Waltham, MA). BSA was used as the protein standard. Absorbance was measured with the plate reader SpectraMax M2 (Molecular Devices, San Jose, CA).

### Western blot

Single retinas were sonicated in lysis buffer (PBS, EDTA and Halt protease inhibitor) and centrifuged for 30 min at 4^o^C. 4x Laemmli buffer (Bio-rad, cat# 1,610,747) containing b-mercaptoethanol was added to the samples and heated for 5 min at 95^o^C. Known amounts of protein (10–20 µg/retina) or protein ladder (cat#1,610,375, Bio-rad, Hercules, CA) were loaded in 4–20% polyacrylamide gels (Bio-Rad #456–1095). Proteins were transferred onto nitrocellulose using the Bio-Rad trans blot turbo transfer system. Membranes were blocked in 2% BSA in TBS-T overnight at 4^o^C. Membranes were incubated in primary antibody (see Table 2) at room temperature with rocking for 2 h. Membranes were washed three times at 1x TBS-T for five min each. Membranes were incubated with secondary antibody (IRDye 800CW Donkey anti-rabbit, #926-32213 or IRDye 680CW Donkey anti-mouse, #926-68022,1:5000 in 1% BSA/TBS) at room temperature for 1 h protected from light. Membranes were washed again three times at 1x TBS-T for five min each. Following washing, blots were imaged with a Bio-Rad ChemiDoc system. Band density was quantified by scanning the blot using Adobe Photoshop. Each band was selected with the same frame and set measurements were used to obtain the gray mean value for each. Band intensity measurements from protein of interest were divided by band intensity measurements of loading control (b-actin). Each experimental group consisted of 5 retinas.

### Quantitative PCR

Retinas were extracted from euthanized mice and placed immediately onto dry ice and stored at ^−^80^o^C until homogenized by hand using 1.5ml-capacity pestles (cat#46C911, Grainger, Nashville, TN). RNA was extracted using a Qiagen RNeasy kit (Valencia, CA) according to manufacturer’s protocol. RNA concentration and purity were measured on a spectrophotometer. First-strand complementary DNA (cDNA) was synthesized from 250 ng of RNA from each sample using the Superscript III First-Strand synthesis system and oligo-dT20 primers (Invitrogen, Waltham, MA). Quantitative PCR (qPCR) was performed using Power SYBR green master mix (Applied Biosystems, Waltham, MA). All primer sequences were obtained from previous studies; we assessed the following: *Prdx6*, *Gpx1*, *Ho-1* and *Sod3* (see Table 3). All qPCR was performed in triplicate using an Applied Biosciences 7300 real-time PCR system (Waltham, MA). The amplification threshold was set using system software. To calculate the expression of genes, we first normalized to the CT of the housekeeping gene, GAPDH. Then, we calculated the negative delta delta CT and normalized the results from all transcripts data to reflect the fold change over the negative delta delta CT of the saline-injected control. Each experimental group had 4–5 retinas.

### Immunohistochemistry

Eyes were embedded in paraffin and sectioned at 10 microns according to previously published methods using the Vanderbilt Vision Research Center histology core [8, 19]. Slides were then warmed on a slide warmer at a medium setting (about 40 ^o^C) for 30 min. Slides were then placed in a rack and went through a series of deparaffinization steps: xylene (10 min), 100% ethanol (10 min), 100% ethanol (5 min), 95% ethanol (5 min), 80% ethanol (5 min), 60% ethanol (5 min), 40% ethanol (5 min). Slides were then placed in coplin jar covered with sodium citrate solution and boiled for 30 min (2.94 g of tri-sodium citrate dehydrate in 1 L of DI water, adjusted to pH of 6.0 and then added 0.5ml of Tween 20). Following boiling, slides were washed twice in 1x PBS for 5 min. Then, slides incubated in sodium borohydride solution (0.05 g sodium borohydride dissolved in 50ml DI water, made fresh every time) at room temperature. Slides were then placed in blocking buffer (500mL 1x PBS, 1.25mL Triton-X, 1.25mL Tween 20, 0.5 g sodium citrate, 11.25 g glycine, 5 g BSA) and 5% normal donkey serum (cat #: D9663, Millipore Sigma, Darmstadt, Germany) for 1 h at room temperature. Slides were washed once with 1xPBS and placed in primary antibody diluted in staining buffer (500mL 1x PBS, 1.25mL Triton X, 1.25 mL Tween 20, 5 g BSA) overnight at 4^o^C in a humidified chamber. The following day, slides were twice washed with 1x PBS for 5 min each. Secondary antibody was diluted in staining buffer and was added to the slides for 2 h at room temperature at 1:200 dilution after spinning for 10 min at 13,000 g. After 2 h, slides were washed twice in 1x PBS for 5 min each. Then, slides were coverslipped with Vectashield containing DAPI (cat#: H-1200-10, Vector Laboratories, Burlingame, CA) and sealed with nail polish. Slides were imaged on a Nikon Eclipse epifluorescence microscope (Nikon, Melville, NY). All images were collected from the same retinal region with identical magnification, gain and exposure settings. Fluorescence intensity was quantified via ImageJ as previously described [8]. A rectangle was selected around the region of interest, channels were split for multiple antibodies, threshold was adjusted, noise was de-speckled and fluorescence intensity was measured. Fluorescence intensity was normalized to saline-injected mice. Each experimental group included 5 eyes.

### Optic nerve counts

Optic nerves were post-fixed in glutaraldehyde followed by Resin 812 embedding and Araldite 502 (cat#: 14,900 and 10,900 respectively, Electron Microscopy Sciences, Hatfield, PA) according to previously published protocols [8, 19, 26]. Leica EM-UC7 microtome was used to collect 1 μm thick sections of the optic nerves. Sections were then stained with 1% paraphenylenediamine and 1% toluidine blue and were imaged on a Nikon Eclipse Ni-E microscope using 100x oil immersion objective (Nikon Instruments, Melville, NY). The optic nerves were montaged into a 5 × 5 image using the Nikon Elements software to scan a large image. We used the Counting Array and Better Cell Counter plugins to ImageJ, which creates a grid of nine squares overtop the montaged optic nerve. We manually counted healthy and degenerating axons, which are color-coded by the plugins. Degenerative axon profiles were identified by dark paraphenylenediamine staining due to collapsed myelin or loose myelin (onioning) surrounding the axon. A grid was used to avoid bias, by always counting in the same squares, using a cross configuration. 20% of the optic nerve cross-sectional area was counted and the total was multiplied by five to estimate total and degenerating axons within the nerve. Each experimental group included 4–5 nerves.

### Data Analysis

All statistical analyses were performed using GraphPad Prism software (La Jolla, CA). A one-way ANOVA with a Bonferroni or Tukey post hoc test (a = 0.05) was used to analyze western blot quantification, IHC fluorescence quantification, ON quantification data, and ERG/VEP latencies and amplitudes. A one-way ANOVA and Dunnett’s multiple comparisons post hoc test (a = 0.05) were used to analyze the qPCR results. Means and standard deviation were calculated for each data set.

**Table 2:** Antibodies used in this study

Antibody	Company	Catalog Number	Species	Dilution for western blot	Dilution for IHC
Nrf2	Abcam	137,550	Rabbit	1:1000	1:200
pNrf2	ThermoFisher	PA5-67520	Rabbit	1:1000	1:200
b-actin	Cell Signaling	E4D9Z	Mouse	1:1000	N/A
RFP (for tdTomato)	ThermoFisher	MA5-15257	Mouse	1:200	1:200
Prdx6	Abcam	133,348	Rabbit	1:1000	N/A
Gpx1	ThermoFisher	PA5-26323	Rabbit	1:500	N/A
SOD3	Abcam	80,946	Rabbit	1:1000	N/A
b-tubulin	Sigma	T8678	Mouse	N/A	1:300

**Table 3:** qPCR primers used in this study

Gene	Forward Primer	Reverse Primer
Prdx6	TTG ATG ATA AGG GCA GGG AC	CTA CCA TCA CGC TCT CTC CC
Gpx1	GGTTCGAGCCCAATTTTACA	CCCACCAGGAACTTCTCAAA
Ho-1	CCTTCCCGAACATCGACAGCC	GCAGCTCCTCAAACAGCTCAA
SOD3	AGGTGGATGCTGCCGAGAT	TCCAGACTGAAATAGGCCTCAAG

## Results

### Knockdown of Nrf2 in either RGCs or glia decreases the endogenous antioxidant response

**Fig. 1:**
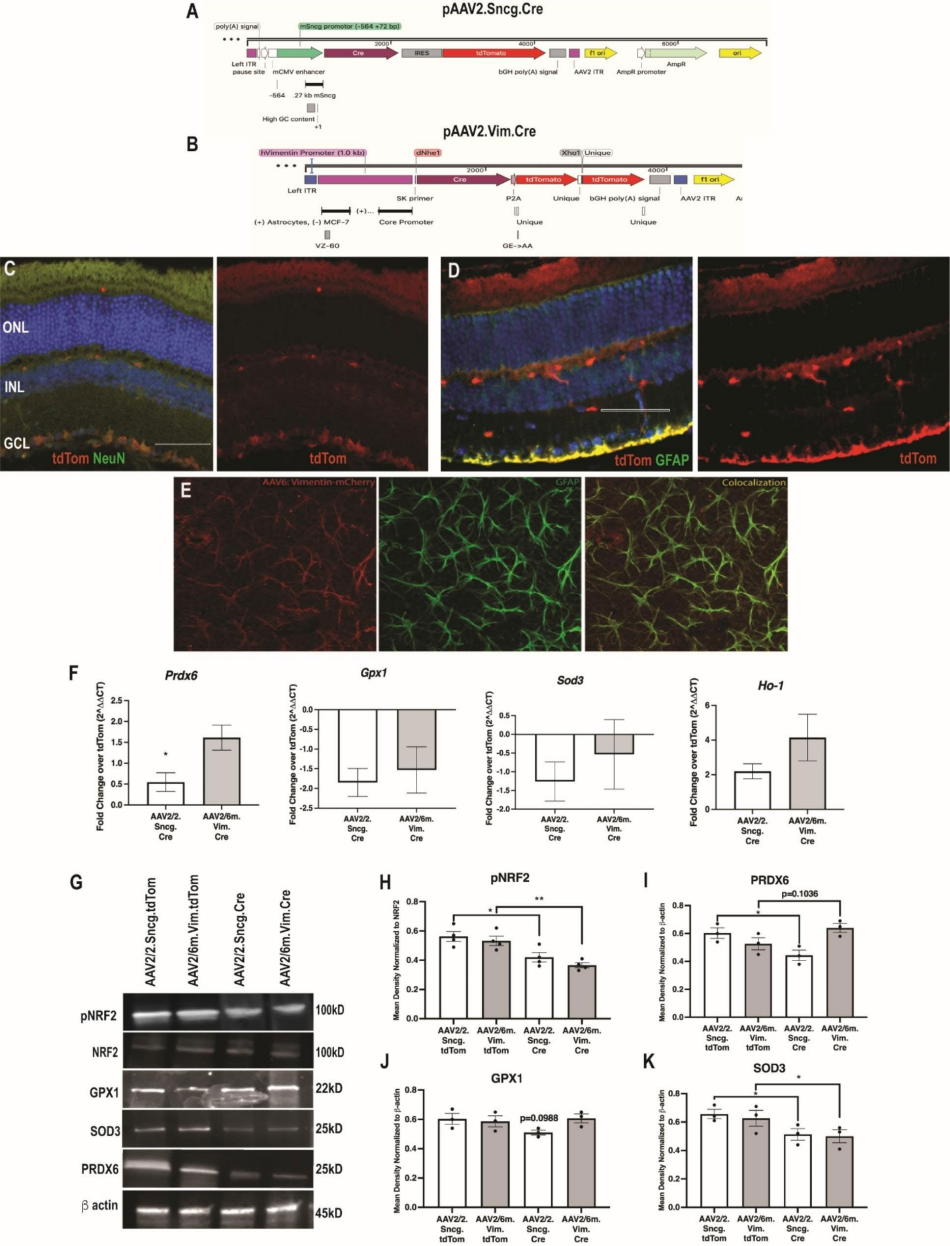
Knockdown of Nrf2 in either RGCs or glia decreases the IOP-induced endogenous antioxidant response. **A, B**) Plasmid maps of pAAV2.Sncg.Cre and pAAV2.hVimentin.Cre, respectively. **C**) Representative fluorescence micrograph of retina from a mouse transduced with AAV2/2.Sncg.Cre.tdTomato (red) and labeled with anti-NeuN (green). **D**) Representative fluorescence micrograph of retina from a mouse transduced with AAV2/6m.Vim.Cre.tdTom (red) and labeled with anti-GFAP (green). Scale bar shown is 50 μm and applies to all micrographs. **E**) Representative confocal micrograph of a flat-mounted retina from a mouse transduced with AAV2/6m.Vim.mCherry and labeled with anti-GFAP (green). **F**) Quantification of *Prdx6, Gpx1, Sod3* and *Ho-1*, all ARE-driven transcripts, at 2 weeks post-IOP elevation in both AAV2/2.Sncg.Cre and AAV2/6m.Vim.Cre treated groups. Fold change was compared to their respective controls, AAV2/2.Sncg.tdTom and AAV2/6m.Vim.tdTom, *p < 0.05. **G**) Representative Western blots for NRF2 and ARE-related proteins. **H**) Quantification for pNRF2 normalized to total NRF2. **I-K**) Quantifications for PRDX6 (I), GPX1 (J), and SOD3 (K) all normalized to b-actin, *p < 0.05, **p < 0.001

In order to explore which cell type, RGCs or glial cells, were responsible for the endogenous antioxidant response after IOP elevation, we knocked down Nrf2 in each cell type separately by injecting appropriate AAVs into Nrf2^fl/fl^ mice. The RGC-directed and glial-directed pAAV constructs are shown in Fig. 1A, B. Intravitreal injection of AAV2/2.Sncg.Cre into Nrf2^fl/fl^ mice resulted in RGC-specific expression based on co-localization with NeuN in the ganglion cell layer (GCL; Fig. 1C). Intravitreal injection of AAV2/6m.Vim.Cre in Nrf2^fl/fl^ mice resulted in glial cell specific expression based on double-labeling with GFAP (Fig. 1D). Because GFAP is expressed by astrocytes and Müller glia in the retina [35–38], co-localization of GFAP and tdTom in retina cross-sections in the GCL accounts for both cell types. However, in retinal flatmounts the morphology demonstrates that we primarily transduced astrocytes (Fig. 1E).

We then quantified levels of representative ARE-driven transcripts that we detected previously as being elevated during the retina’s endogenous antioxidant response to elevated IOP and for which good antibodies exist [14]. All mice were injected with microbeads to elevate IOP two weeks after injection with the respective AAVs and tissue was collected after two weeks of elevated pressure. Results were compared to Nrf2^fl/fl^ mice injected with the same serotype AAV and promoters, delivering tdTom without Cre and referenced as fold change over tdTom (Fig. 1F). The previously reported IOP-induced increase in Prdx6 [14] was blunted by knock-down of Cre in the RGCs, but was unaffected by knock-down of Cre in the glia. The difference in Prdx6 expression between mice treated with AAV2/2.mSncg.tdTomato and those treated with AAV2/2.mSncg.Cre was statistically significant (p = 0.0294; n = 4 retinas/group; Fig. 1F). Knock-down of Nrf2 in either the RGCs or glia prevented the IOP-induced increase in expression of Gpx1 and Sod3, but neither affected the increase in Ho-1 (p = 0.6651, p = 0.533, and p = 0.1775 respectively; n = 3–4 retinas/group respectively; Fig. 1F).

To confirm that NRF2 activation was decreased in the AAV.Cre groups, we quantified the ratio of phosphorylated to total NRF2 (Fig. 1G, H). There was less phosphorylated NRF2 in both the AAV2/2.Sncg.Cre and the AAV2/6m.Vim.Cre injected mice than in their respective controls (p = 0.0227 and p = 0.008, respectively; n = 4 retinas/group).

We also quantified the endogenous antioxidant response at the protein level (Fig. 1G-K). Consistent with the PCR data, knock-down of Nrf2 in the RGCs prevented the IOP-induced increase in PRDX6, but knock-down in the glia had no effect (p = 0.0395; n = 3 retinas/group; Fig. 1I). There was no statistically significant difference in GPX1 levels in either group (p = 0.1852; n = 4 retinas/group; Fig. 1J). Lastly, there was less SOD3 in both the RGC-specific and the glial-specific knockdown groups in comparison to their respective controls (p = 0.0117 and p = 0.0231, respectively; n = 3 retinas/group; Fig. 1K).

### Knockdown of Nrf2 in either RGCs or glia causes earlier onset vision loss and axon degeneration

**Fig. 2:**
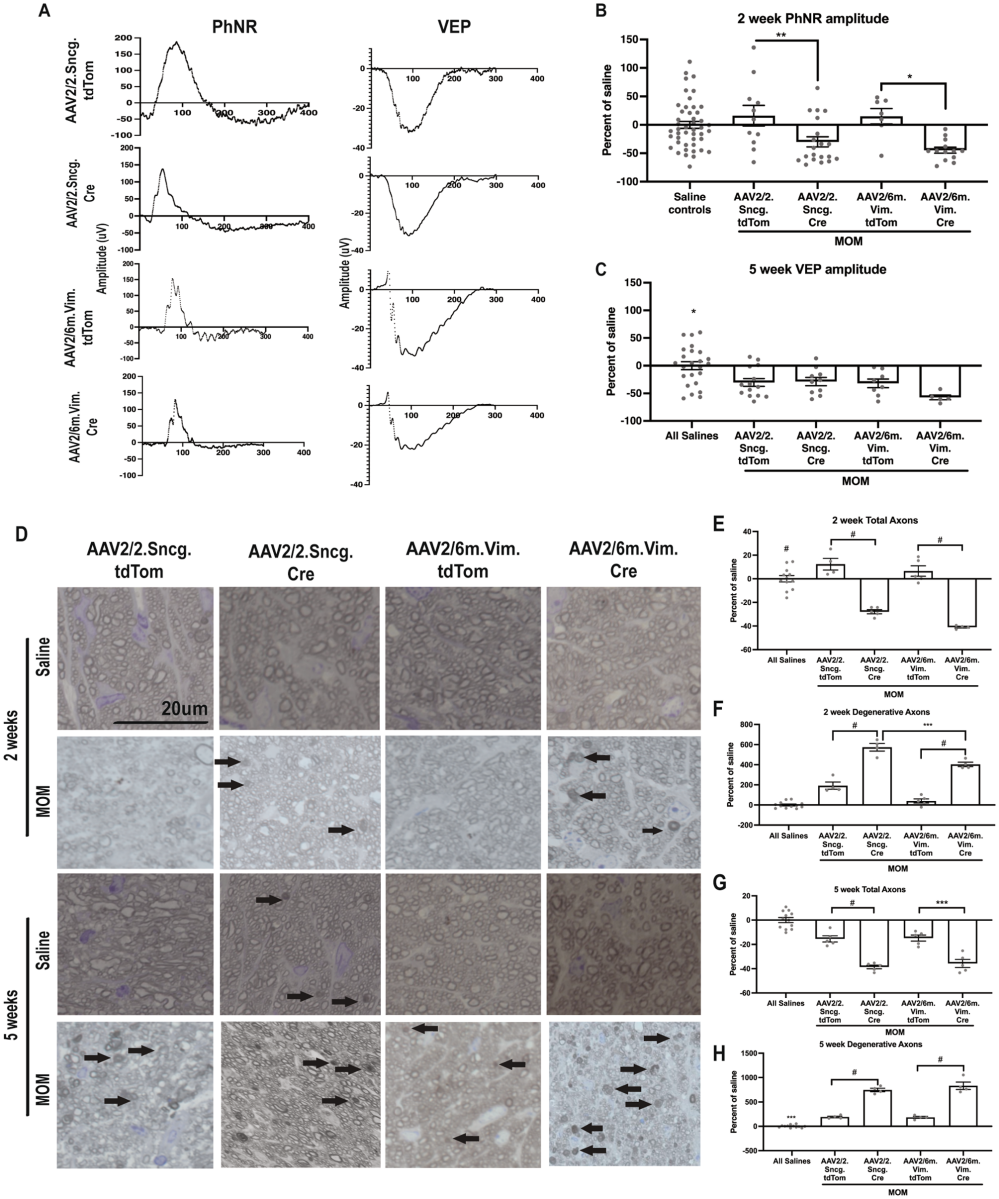
Knockdown of Nrf2 in either RGCs or astrocytes decreases visual function and accelerates axon degeneration. **A**) Representative waveforms for PhNR and VEP N1 in all experimental groups. **B**) Quantification of PhNR amplitudes in all experimental groups. **C**) Quantification of VEP N1 amplitudes in all experimental groups, with AAV2/6m.Vim.Cre group having the lowest amplitude in comparison to all other groups. *p < 0.05. **D**) Representative brightfield micrographs of optic nerves at 2- and 5-wks post-saline injection or IOP elevation in all experimental groups, with black arrows indicating degenerative axons. Scale bar applies to all micrographs. **E**) and **F**) Quantification of total and degenerative axons at 2 weeks. **G**) and **H**) Quantification of total and degenerative axons at 5 weeks. *p < 0.05, **p < 0.01, ***p < 0.001, ****p < 0.0001

We previously detected PhNR deficits at 2-wks post-IOP elevation in the microbead occlusion model [8]. There was no difference between saline groups with or without delivery of Cre, therefore, all saline groups were combined for calculating percent of saline. Nrf2 knockdown in either the RGCs (n = 20) or the glial cells (n = 13) further decreased the PhNR amplitude at 2- weeks post-IOP elevation compared to saline controls (n = 46) (p = 0.036 and p = 0.0013, respectively; Fig. 2A, B). The PhNR amplitudes also were decreased compared to the no-Cre, microbead injected controls; p = 0.0195 (n = 11) and p = 0.0041 (n = 7) for the RGC or glia-directed vectors, respectively. At 5-wks post-IOP elevation, there was no difference in PhNR amplitude between the AAV2/6.Vim.Cre and respective no-Cre groups (p = 0.5182, data not shown). In contrast, RGC-specific knockdown of Nrf2 caused a further reduction in PhNR amplitude compared to no-Cre controls (data not shown).

Knock-down of Nrf2 in either cell-type had no effect on the VEP at 2-wks post-IOP elevation as expected (data not shown). At 5-wks post-IOP elevation the VEP amplitude was similarly reduced in all MOM mice compared to saline controls (n = 24) regardless of Cre delivery (p < 0.05; n = 14 and 8 eyes/group for RGC and glial-directed non-Cre controls and n = 10 and 5 for the RGC and glial-directed Cre groups, respectively; Fig. 2A, C).

More degenerative axon profiles were evident in optic nerve cross-sections from the AAV.Cre injected mice compared to controls at both 2- and 5-wks post-IOP elevation (Fig. 2D). There was no statistically significant difference in axon counts between the no-Cre and Cre saline injected mice so all saline groups were combined. Fewer total axons (p < 0.0001) and more degenerative axons (p < 0.0001 at both time points) were present in optic nerves from AAV2/2.Sncg.Cre injected mice compared to saline controls (n = 5 and 12 nerves/group, respectively; Fig. 2E-H). Similarly, AAV2/6.Vim.Cre injected mice had fewer total axons (p < 0.0001; n = 4) and more degenerative axons (p < 0.0001 at both time points) than saline control mice at both time-points (Fig. 2D-H). There was no statistically significant difference in the total number of axons between the AAV2/2.Sncg.Cre and AAV2/6.Vim.Cre groups (n = 5 and 4 nerves/group, respectively; Fig. 2E, G). However, there were significantly more degenerative axons in the AAV2/2.Sncg.Cre mice compared to AAV2/6.Vim.Cre mice at 2-weeks (p = 0.0005). This effect was not present at 5-weeks.

### Activation of the ARE in RGCs at 2 weeks post-IOP elevation

**Fig. 3:**
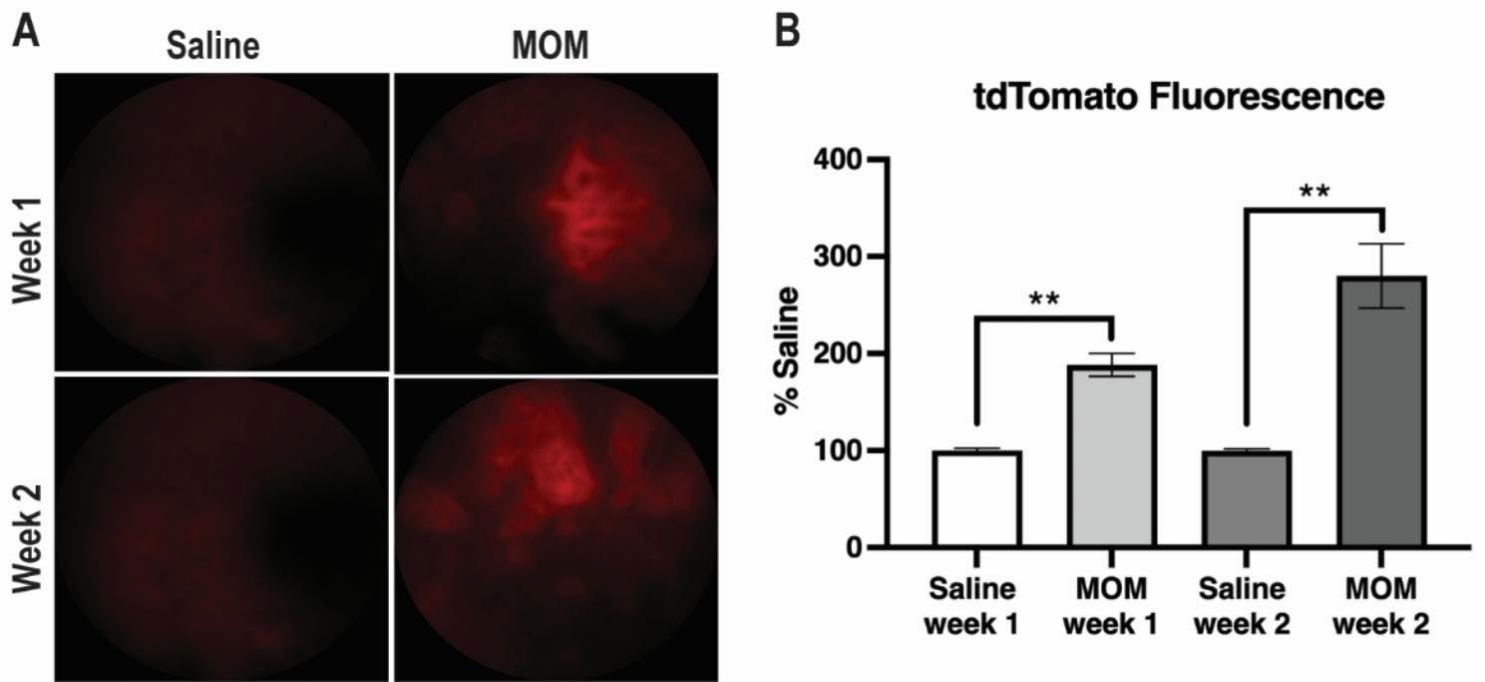
ARE is activated in RGCs in glaucomatous retinas. **A**) Representative fundus images of tdTom fluorescence in control and IOP-elevated mice injected with AAV2/2.Trx.ARE.tdTomato.SV40-zsGreen. **B**) Quantification of tdTom fluorescence showing an increase at 1- and 2-weeks post-IOP elevation. **p < 0.01

As an independent approach to determine if elevated IOP can activate the ARE in the RGCs, we intravitreally injected an AAV2/2 delivering ARE driving tdTom into wildtype mice two weeks prior to inducing elevated IOP. The resulting tdTom fluorescence was imaged in vivo at 1- and 2-weeks post-IOP elevation (Fig. 3A). Retinal fluorescence was evident in both MOM groups, but not in the saline controls (Fig. 3A). This corresponded to an approximately 2-fold increase at 1-wk and 3-fold increase at 2-wks (p = 0.0015 and p = 0.0025, respectively; n = 4-6eyes/group; Fig. 3B). The greater increase at 2-wks compared to 1-wk was statistically significant (p = 0.029; n = 6 eyes/group). The greater ARE activation at 2-wks matched our previously findings of peak activation of the endogenous antioxidant response at 2-wks post-IOP elevation [14].

### Expression of Nrf2 in ganglion cell layer (GCL) neurons in Nrf2 KO mice

**Fig. 4:**
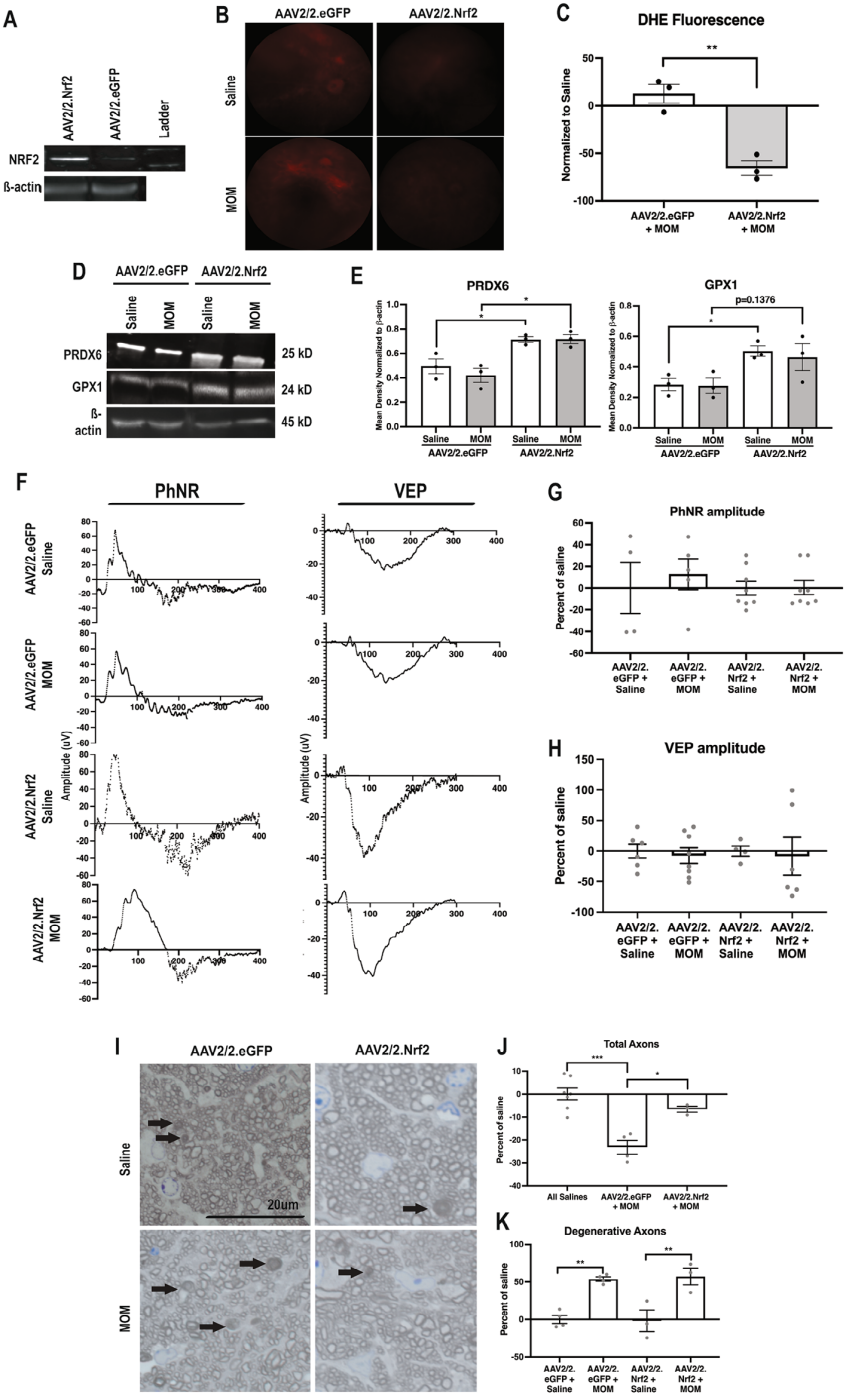
Nrf2 overexpression in GCL neurons in Nrf2 KO mice decreases ROS, preserves visual function and optic nerve histology. **A**) Representative Western blot for NRF2 normalized to b–actin showing increased levels of NRF2 in AAV2/2.Nrf2 injected retinas in comparison to AAV2/2.eGFP injected controls. Representative fundus images (**B**) and quantification (**C**) of DHE fluorescence in all groups, *p < 0.05, **p < 0.001. Representative Western blots (**D**) and quantification (**E**) of PRDX6, GPX1 normalized to b–actin, *p < 0.05. Representative PhNR and VEP waveforms (**F**) and quantifications of amplitudes (**G, H**) at 2 weeks post-IOP elevation. **I**) Representative brightfield micrographs of optic nerves, arrows indicate degenerative axons. Scale bar applies to all micrographs. Quantification of total (**J**) and degenerative (**K**) axons in all groups. *p < 0.05, **p<0.001, ***p < 0.0001 and ****p < 0.00001

We previously demonstrated that Nrf2 KO mice with elevated IOP lack an endogenous retinal antioxidant response and exhibit earlier onset of axon degeneration and vision loss [8]. To determine if expression of Nrf2 only in the GCL neurons would be sufficient to activate the antioxidant response, we intravitreally injected AAV2/2.Nrf2 or AAV2/2.eGFP into Nrf2 KO mice and assessed all outcomes at 2 weeks post-IOP elevation. Total retinal NRF2 levels were increased in AAV2/2.Nrf2 injected eyes but not in controls (Fig. 4A). Treatment with AAV2/2.Nrf2 decreased DHE fluorescence regardless of IOP elevation (Fig. 4B, C). In both the saline-injected control mice and microbead-injected mice, treatment with AAV2/2.Nrf2 increased PRDX6 levels (p = 0.0276 and p = 0.0121, respectively; n = 3 retinas/group; Fig. 4D, E). Similarly, GPX1 levels were also increased (p = 0.0141 compared to saline-injected controls and p = 0.1376 compared to microbead-injected mice; n = 3 retinas/group; Fig. 4D, E). There was no difference in SOD3 levels between groups (data not shown).

We then assessed if transduction of the RGCs with AAV2/2.Nrf2 was sufficient to prevent vision loss. We previously published that the PhNR and VEP amplitudes were reduced in wildtype, but not Nrf2 KO microbead-injected mice compared to their relevant controls [8]. Here we detected no change in PhNR amplitude between saline and microbead injected Nrf2 KO mice treated with AAV2/2.eGFP, similar to our previous findings [8]. The PhNR amplitude in Nrf2 KO mice injected with saline and treated with AAV2/2.Nrf2 was increased compared to saline-injected AAV2/2.eGFP controls (p = 0.0357; n = 6–8 eyes/group). Thus, we calculated the percent of saline for each microbead injected group to their own saline controls. Elevated IOP did not affect the PhNR amplitude in the Nrf2 KO mice treated with AAV2/2.Nrf2 (n = 6–8 eyes/group; Fig. 4F, G). Similarly, treatment with AAV2/2.Nrf2 increased the VEP N1 amplitude in Nrf2 KO mice whether they were injected with saline or microbeads (p = 0.0015 and p = 0.0146, respectively; n = 6–8 eyes/group respectively; Fig. 4F, H). Therefore, the percent of saline was calculated based on the saline control for the corresponding microbead injected group. There was no difference in VEP N1 amplitude between saline and microbead injected Nrf2 KO mice treated with AAV2/2.Nrf2 (Fig. 4F, H).

In agreement with our previous study in microbead occluded Nrf2 KO mice, we detect fewer total axons and more axon degeneration in Nrf2 KO mice treated with AAV2/2.eGFP after microbead occlusion as compared to saline-injected controls at 2-weeks post-IOP elevation (p = 0.0002 and p = 0.0031; n = 3–4 nerves/group; Fig. 4I-K) [8]. Treatment with AAV2/2.Nrf2 had no effect on the total number of axons in the saline injected mice, therefore, all saline controls were combined. Microbead-injected, Nrf2 KO mice treated with AAV2/2.Nrf2 had more total axons compared to those treated with AAV2/2.eGFP (p = 0.0113; n = 3–4 nerves/group; Fig. 4I, J). Treatment with AAV2/2.Nrf2 decreased the number of degenerative axons in the saline controls (p = 0.00085), therefore, each microbead group was normalized to its own saline control group. Microbead-injected, Nrf2 KO mice treated with AAV2/2.Nrf2 had fewer degenerative axons compared to those treated with AAV2/2.eGFP, but upon normalization to their own saline groups this effect was lost (Fig. 4I, K). Notably, the Nrf2 knockout AAV2/2.Nrf2 treated, saline injected group had an average of 132 ± 33 (sd) degenerative axons (n = 3) and the corresponding microbead injected group had an average of 180 ± 26 (sd) degenerative axons (n = 3), using the student’s t-test this yields a p value of 0.03, suggesting a protective effect of Nrf2 that is not obvious in the normalized data.

### Overexpression of Nrf2 in the GCL neurons of wild-type mice increases the endogenous antioxidant response after IOP elevation

**Fig. 5:**
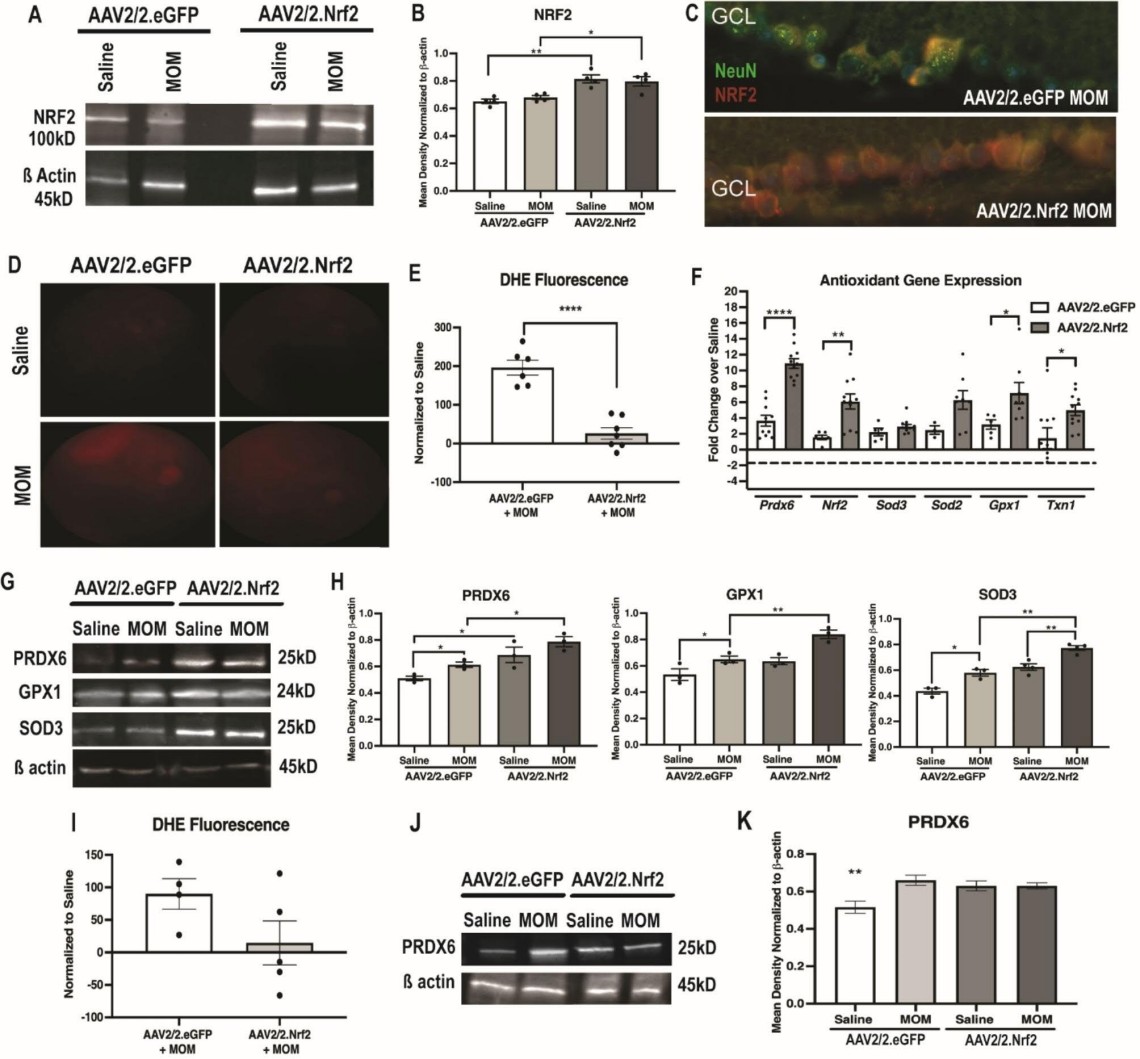
Overexpression of Nrf2 in GCL neurons in WT mice increases endogenous antioxidant response of the retina at 2 and 5 weeks post-IOP elevation. Representative Western blots (**A**) and quantification (**B**) for NRF2 normalized to b-actin, *p < 0.05, **p < 0.001. **C**) Representative fluorescence micrographs of retina GCL from mice with elevated IOP and treated with either AAV2/2.eGFP or AAV2/2.Nrf2 and labeled with anti-NRF2 (red) and anti-NeuN (green). Representative fundus images (**D**) and quantification (**E**) of DHE fluorescence in all groups at 2 wks post-IOP elevation, ****p < 0.00001. **F**) Quantification of antioxidant gene transcription (*Prdx6, Nrf2, Sod3, Sod2, Gpx1* and *Txn1)* shown as fold change over appropriate saline controls at 2 wks post-IOP elevation. Representative Western blots (**G**) and quantifications (**H**) for PRDX6, GPX1, and SOD3 all normalized to b-actin at 2 wks post-IOP elevation, *p < 0.05, **p < 0.001. **I**) Quantification of DHE fluorescence in both AAV2/2.eGFP and AAV2/2.Nrf2 mice at 5 wks post-IOP elevation in comparison to saline-injected controls, *p < 0.05. Representative western blots (**J**) and quantification (**K**) for PRDX6 normalized to b-actin at 5 wks post-IOP elevation, **p < 0.001

We sought to determine if overexpression of Nrf2 in GCL neurons of wildtype mice would enhance the retina’s endogenous response to elevated IOP. Treatment with AAV2/2.Nrf2 increased total retina levels of NRF2 in both saline and microbead-injected mice (p = 0.0028 and p = 0.0184; n = 4 retinas/group; Fig. 5A, B). We confirmed localization of the increased NRF2 in the GCL neurons by co-labeling with NeuN (Fig. 5C). DHE fluorescence was increased in the microbead-injected AAV2/2.eGFP mice in comparison to their saline-injected controls (p = 0.0012; n = 6–8 eyes/group; Fig. 5D, E). Treatment with AAV2/2.Nrf2 prevented the IOP-induced increase in DHE fluorescence (p = 0.2767; n = 6–8 eyes/group; Fig. 5D, E).

Levels of antioxidants at the mRNA and protein level in IOP-elevated mice were further increased by treatment with AAV2/2.Nrf2. *Prdx6, Nrf2, Gpx1* and *Txn1* were increased after elevation of IOP and treatment with AAV2/2.Nrf2 in comparison to the AAV2/2.eGFP controls (p < 0.0001, p = 0.0035, p = 0.0467, and p = 0.0237, respectively; n = 5 retinas/group; Fig. 5F). This was above and beyond the endogenous increase in these antioxidant proteins we previously reported [8]. In AAV2/2.eGFP mice, PRDX6, GPX1 and SOD3 were increased in microbead-injected mice at 2 wks post-IOP elevation in comparison to saline-injected controls (p = 0.0179, p = 0.0377, and p = 0.014 respectively; n = 3–4 retinas/group; Fig. 5G, H). This was consistent with our previous studies, which indicated that the retina endogenously responds to increased ROS by upregulating expression of these proteins [8]. Mice that received AAV2/2.Nrf2 had an even greater increase in these antioxidant proteins in both saline and microbead groups than AAV2/2.eGFP controls (Fig. 5G, H). PRDX6 levels were increased in AAV2/2.Nrf2 treated saline or microbead-injected controls in comparison to AAV2/2.eGFP injected mice (p = 0.043 and p = 0.0153, respectively; n = 3–4 retinas/group; Fig. 5G, H). GPX1 was also increased in AAV2/2.Nrf2 treated saline or microbead-injected controls in comparison to their AAV2/2.eGFP treated counterparts (p = 0.0124 and p = 0.0096, respectively; n = 3–4 retinas/group; Fig. 5G, H). Similarly, SOD3 levels were increased in AAV2/2.Nrf2 treated saline-injected controls in comparison to their AAV2/2.eGFP treated counterparts (p = 0.0032 and p = 0.0013, respectively; n = 3–4 retinas/group; Fig. 5G, H).

DHE fluorescence was still elevated in AAV2/2.eGFP mice injected mice in comparison to saline-injected controls at 5-weeks post-IOP elevation (p = 0.0112; n = 4–5 eyes/group; Fig. 5I). In contrast, there was no increase in DHE fluorescence in the AAV2/2.Nrf2 injected mice (p = 0.7042; n = 4–6 eyes/group; Fig. 5I). There was no additional increase in PRDX6 levels upon treatment with AAV2/2.Nrf2 at this time point (n = 5 retinas/all groups; Fig. 5J,K).

### Overexpression of Nrf2 in GCL neurons protects against vision loss and axon degeneration at 2 and 5 weeks post-IOP elevation

**Fig. 6:**
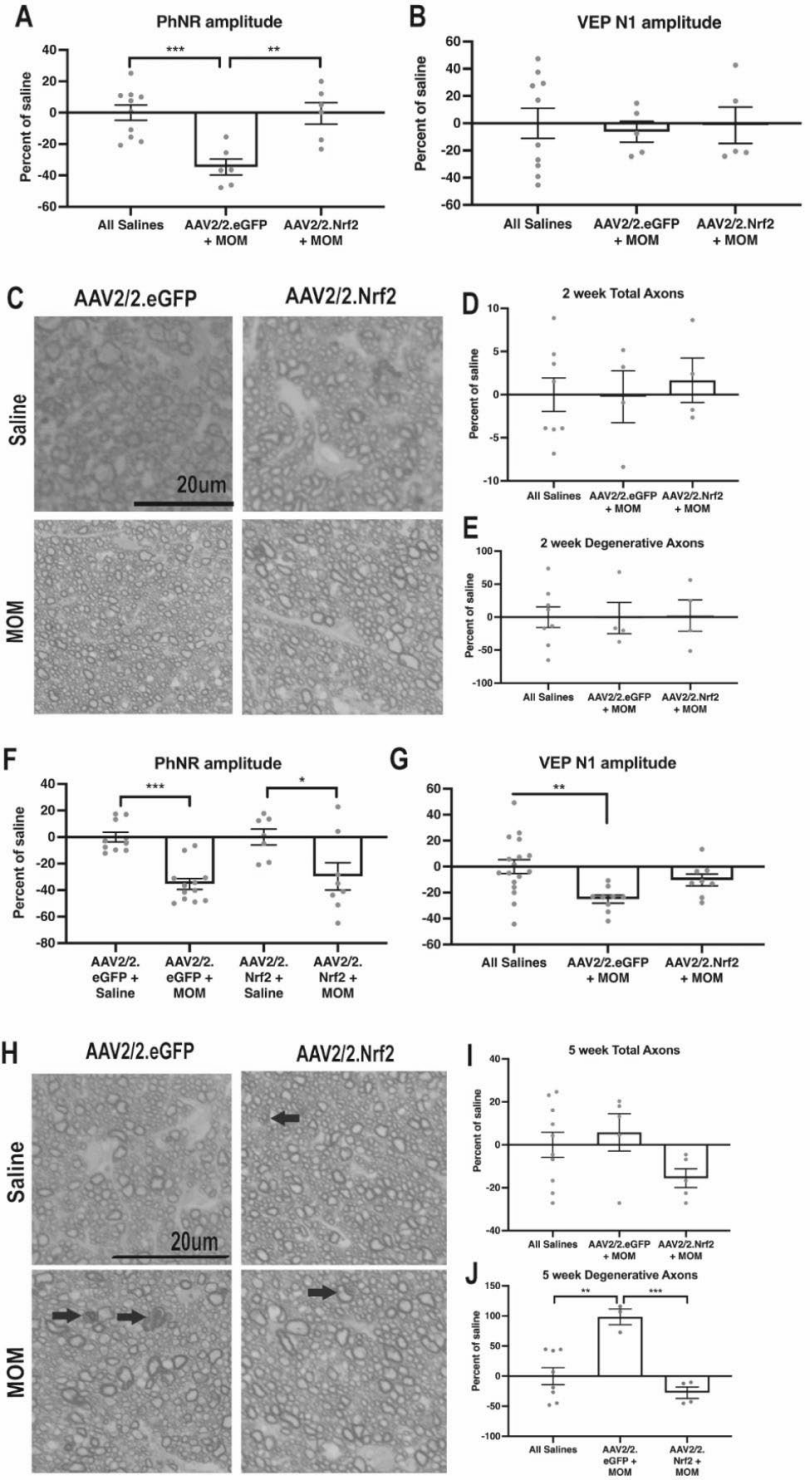
Overexpression of Nrf2 in GCL neurons in WT mice preserves visual function and protects RGCs at 2 and 5 wks post-IOP elevation. **A**) Quantification of PhNR amplitudes in AAV2/2.eGFP and AAV2/2.Nrf2 groups at 2 wks post-IOP elevation, **p < 0.001. **B**) Quantification of VEP N1 amplitudes in AAV2/2.eGFP and AAV2/2.Nrf2 groups at 2 wks post-IOP elevation, showing no differences between any groups. **C**) Representative brightfield micrographs of AAV2/2.eGFP and AAV2/2.Nrf2 optic nerves at 2 wks post-IOP elevation or post-saline injection. Scale bar applies to all micrographs. Quantification of total (**D**) and degenerative (**E**) axons at 2 wks post-IOP elevation, showing no differences in any groups. **F**) Quantification of PhNR amplitude in AAV2/2.eGFP and AAV2/2.Nrf2 groups at 5 wks post-IOP elevation, *p < 0.05, ***p < 0.0001. **G**) Quantification of VEP N1 amplitudes in AAV2/2.eGFP and AAV2/2.Nrf2 groups at 5 wks post-IOP elevation, ***p < 0.0001. **H**) Representative brightfield micrographs of AAV2/2.eGFP and AAV2/2.Nrf2 optic nerves at 5 wks post-IOP elevation or post-saline injection. Scale bar applies to all micrographs. Arrows point to degenerative axons. Quantification of total (**I**) and degenerative (**J**) axons in all groups at 5 wks post-IOP elevation, *p < 0.05, **p < 0.001, ***p < 0.001

As expected, at 2-wks post-IOP elevation, the PhNR amplitude was decreased in AAV2/2.eGFP mice in comparison to saline controls (p = 0.0008; n = 6 mice/group; Fig. 6A), matching our previous results [8]. There was no statistically significant difference in PhNR amplitude between the saline groups so they were combined for analyses. Treatment with AAV2/2.Nrf2 prevented this decrease (n = 6 mice/group; Fig. 6A). When normalized to saline-injected controls, microbead-injected, AAV2/2.Nrf2 treated mice showed significantly greater PhNR amplitude in comparison to microbead-injected, AAV2/2.eGFP treated mice (p = 0.0026; Fig. 6A). There was no significant difference in the PhNR latencies of any of the experimental groups (data not shown). As expected, that there were no differences in VEP N1 amplitudes in saline groups or at 2 wks post-IOP elevation (n = 5 mice/group; Fig. 6B). There were also no differences in total or degenerative axons between groups, all optic nerves appeared normal (Fig. 6C-E).

At 5-wks post-IOP elevation, both microbead-injected groups had a reduced PhNR amplitude as compared to their relevant saline controls (p = 0.0004 (n = 10–12) and p = 0.0173 (n = 7–8) for AAV.eGFP and AAV.Nrf2 groups, respectively; Fig. 6F). Further, there was no difference between the AAV2/2.Nrf2 and AAV2/2.eGFP treated microbead-injected mice after normalization to saline controls (Fig. 6F). This suggests that the protective effect of AAV2/2.Nrf2 on the PhNR was lost over time. As expected, the VEP N1 amplitude was decreased in the microbead-injected AAV.eGFP mice (n = 9) in comparison to saline-injected controls (n = 17; p = 0.005; Fig. 6G). In contrast, there was no statistically significant difference between saline controls and the microbead-injected mice treated with AAV2/2.Nrf2 (n = 8; Fig. 6G). Degenerative axons were present in glaucomatous optic nerves at 5-wks post-IOP elevation (Fig. 6H). There was no statistically significant difference in the total number of axons between the saline groups or the combined saline treated mice (n = 10) and any of the microbead injected groups (n = 5/group; Fig. 6I). There was also no statistically significant difference in the number of degenerative axons between microbead-injected, AAV2/2.Nrf2 treated mice (n = 4) and all saline groups combined (n = 8/group; Fig. 6J). The number of degenerative axons at 5 weeks in the AAV2/2.eGFP microbead injected group (n = 3) was greater than the saline controls, p = 0.0024, and the AAV2/2.Nrf2 treated, microbead injected mice (n = 4), p = 0.0009 (Fig. 6J).

## Discussion

We previously discovered that an Nrf2-mediated endogenous antioxidant response of the retina delays the onset of glaucomatous neurodegeneration [8]. In this study, we sought to elucidate the roles of the RGCs and the glial cells in this response and to assess the efficacy of AAV2/2.Nrf2 as a neuroprotective treatment. Transduction with AAV2/2.CMV.Nrf2 in the Nrf2 KO background decreased superoxide levels, activated the Nrf2/ARE pathway, and decreased optic nerve pathology. Notably, since the CMV promoter was used for these overexpression experiments it feasible that some displaced amacrine cells and glia were transduced in addition to RGCs [33–35].

To determine if elevated IOP caused activation of the ARE in the RGCs, we transduced the cells with an ARE reporter construct. Activation of the ARE was detected, supporting the hypothesis that the RGCs contribute to the endogenous antioxidant response of the retina to elevated IOP. The increased tdTom fluorescence occurred concurrently with the previously characterized activation of Nrf2 at 2 weeks post-IOP elevation [8].

As another approach, we used different AAV serotype and promoter combinations to specifically deliver Cre to either RGCs or astrocytes in the Nrf2^fl/fl^ mouse. The use of AAV2/2 and the gamma synuclein promoter provided specific expression in the RGCs as previously reported [22]. The use of the AAV6 capsid with tyrosine mutations and a 1 kb vimentin promoter resulted in expression in glial cells. More degenerative axons were detected in the mice with RGC-specific knockdown of Nrf2 than in the mice where Nrf2 knockdown was targeted to the glia. This could be due to a greater role of the RGCs or a difference in level of knockdown within the cells in the two groups. Regardless, either RGC-specific or glial-specific knockdown of Nrf2 resulted in a decreased endogenous antioxidant response, increased glaucomatous pathology and increased vision loss, as well as an earlier onset of this pathology. The ARE reporter results show that the RGCs contribute to the endogenous antioxidant response and that increased Nrf2 in these cells can enhance this response and provide neuroprotection. The data suggests that both neurons and glia contribute to the retina’s endogenous antioxidant response but the amplification of this pathway in RGCs is sufficient to compensate for lack of Nrf2 elsewhere. It is possible that activation of the Nrf2/ARE pathway is important in both glial cells and RGCs for the IOP-dependent antioxidant response, or these two cell types communicate so substantially that this in vivo approach was insufficient to isolate effects from the RGCs or the glial cells independently. Future in vitro experiments may be needed to separate the two potential explanations.

We found that Nrf2 overexpression in GCL neurons, beginning prior to IOP elevation, counteracts the increase in ROS and protects RGCs at both 2 and 5 weeks post-IOP elevation. Our findings agree with previous publications showing an essential role for Nrf2 in neurons’ antioxidant response [13–15]. In the future, it would be interesting to determine the therapeutic window for Nrf2 gene therapy. Additionally, the efficacy of Nrf2 activators should be tested. Pharmacological activation of Nrf2 has been shown to be neuroprotective in a model of ischemia/reperfusion retinal injury [13]. Four Nrf2 activators are currently being explored in clinical trials, which could be used in glaucoma studies in the future [27]. Overexpression or chronic activation of Nrf2 could also have negative side effects. In *Drosophila*, prolonged activation of Nrf2 can shorten lifespan [28]. Additionally, many activators of Nrf2 work by modifying Keap1 so that it cannot degrade Nrf2 via disruption of its cysteine residues—these activators are often nonspecific and can have off-target effects [27, 29]. Therefore, future studies investigating the downstream responses of activation of the Nrf2/ARE pathway might yield more specific therapeutic targets. For example, our work suggests that PRDX6 and SOD3 might contribute significantly to the endogenous antioxidant response, and therefore, exogenously increasing levels of these proteins might be beneficial in delaying or decreasing glaucoma pathogenesis. Previous studies have shown that antioxidants are effective in preserving RGCs and visual function in models of glaucoma [6–8, 30]. This study further supports this approach and provides insights into the best antioxidants to pursue for clinical translation.

Overall, our study elucidates the importance for NRF2 in both RGCs and astrocytes for the retina’s endogenous antioxidant response to glaucoma-induced ROS. Additionally, we show that overexpression of Nrf2 in GCL neurons of wildtype mice is a viable therapeutic approach for axon degeneration, RGC function and other glaucomatous pathology. In contrast, overexpression of Nrf2 in RGCs in an Nrf2 knock-out background is not sufficient to mitigate inevitable glaucomatous pathology.

### Electronic supplementary material

Below is the link to the electronic supplementary material.

**Supplemental Figure 1.** ARE reporter is activated in ARPE-19 cells exposed to an NRF2 activator. A) Plasmid map of pAAV2.Trx.ARE.tdTomato.SV40-HA-zsGreen. B) Sequence of WT ARE and M4 ARE, red letters indicate the mutations made in M4 in comparison to WT ARE. C) Representative fluorescence micrographs of ARPE-19 cells transfected with WT ARE or M4 ARE and treated with DMSO (control) or 5OM sulforaphane (SF), which is known to activate the ARE. White arrows indicate co-labeling of tdTom (red) and zsGreen (green). D) Representative western blot of tdTom (110kd) and ZsGreen (doublet bands at 100kD) of ARPE-19 cells.

**Supplemental Figure 2.** IOP was elevated in microbead injected mice. A, B) IOPs from mice used to generate data shown in Figures 1 and 2. Mice were reinjected with microbeads on day 19. C) IOPs from mice used to generate data shown in Figure 4. D) IOPs from mice used to generate data shown in Figure 6.

## Data Availability

All data generated and analyzed during this study are included in this published article and its supplementary information files.

## Abbreviations

IOP: intraocular pressure
ROS: reactive oxygen species
NRF2: Nuclear factor-erythroid factor 2-related factor 2
ARE: antioxidant response element
RGC: retinal ganglion cell
AAV: adeno-associated virus
PhNR: photopic negative response
KEAP1: Kelch-like ECH associated protein 1
