# Supplementary figures and images for: NRF2/ARE mediated antioxidant response to glaucoma: role of glia and retinal ganglion cells
Source: Acta Neuropathol Commun. 2023 Oct 24;11:171. doi: 10.1186/s40478-023-01663-1 (PMC10594672; doi:10.1186/s40478-023-01663-1)

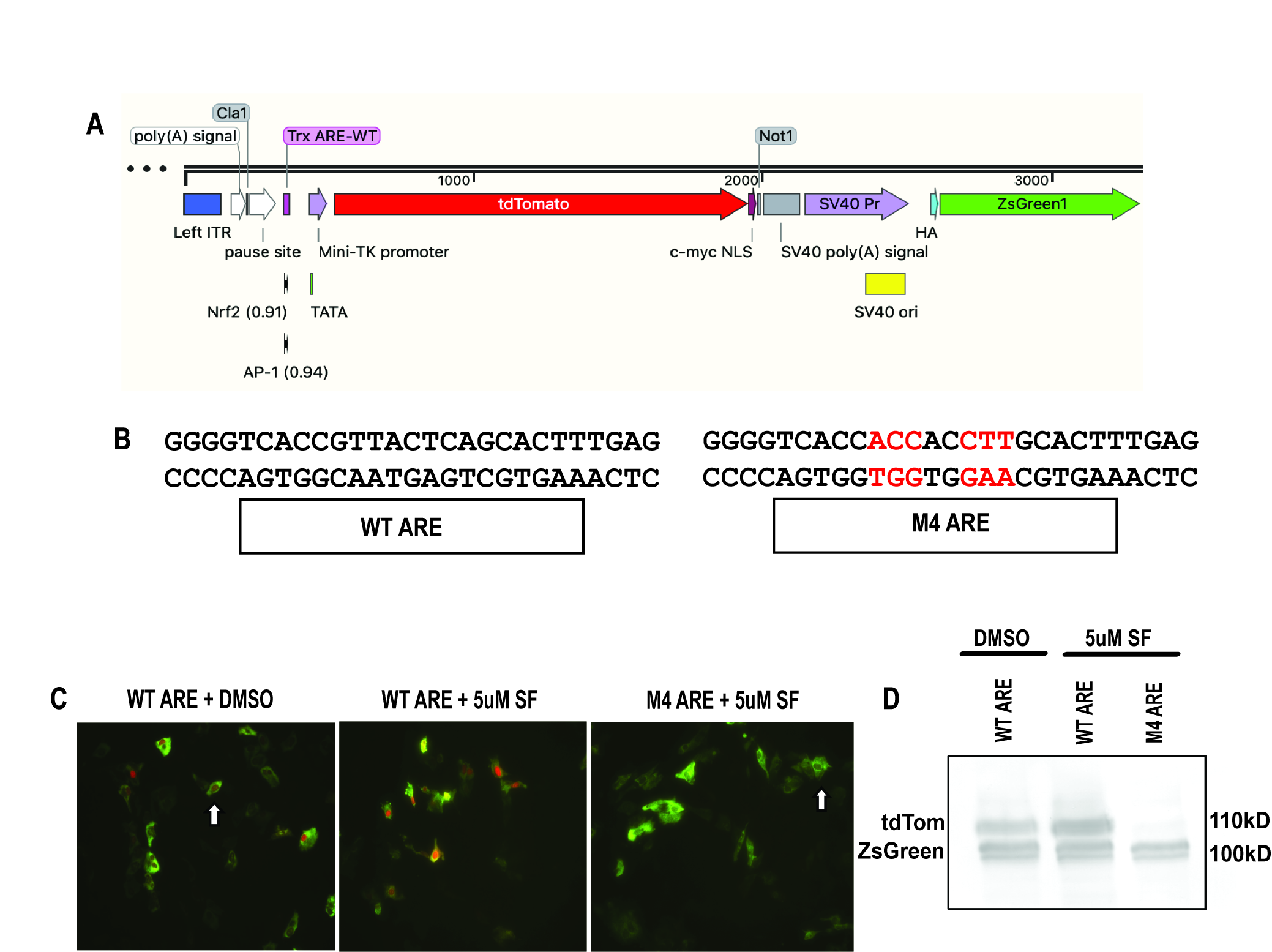

Supplement: Supplementary file 1 — Supplemental Figure 1. ARE reporter is activated in ARPE-19 cells exposed to an NRF2 activator. A) Plasmid map of pAAV2.Trx.ARE.tdTomato.SV40-HA-zsGreen. B) Sequence of WT ARE and M4 ARE, red letters indicate the mutations made in M4 in comparison to WT ARE. C) Representative fluorescence micrographs of ARPE-19 cells transfected with WT ARE or M4 ARE and treated with DMSO (control) or 5OM sulforaphane (SF), which is known to activate the ARE. White arrows indicate co-labeling of tdTom (red) and zsGreen (green). D) Representative western blot of tdTom (110kd) and ZsGreen (doublet bands at 100kD) of ARPE-19 cells. [file 40478_2023_1663_MOESM1_ESM.tif]

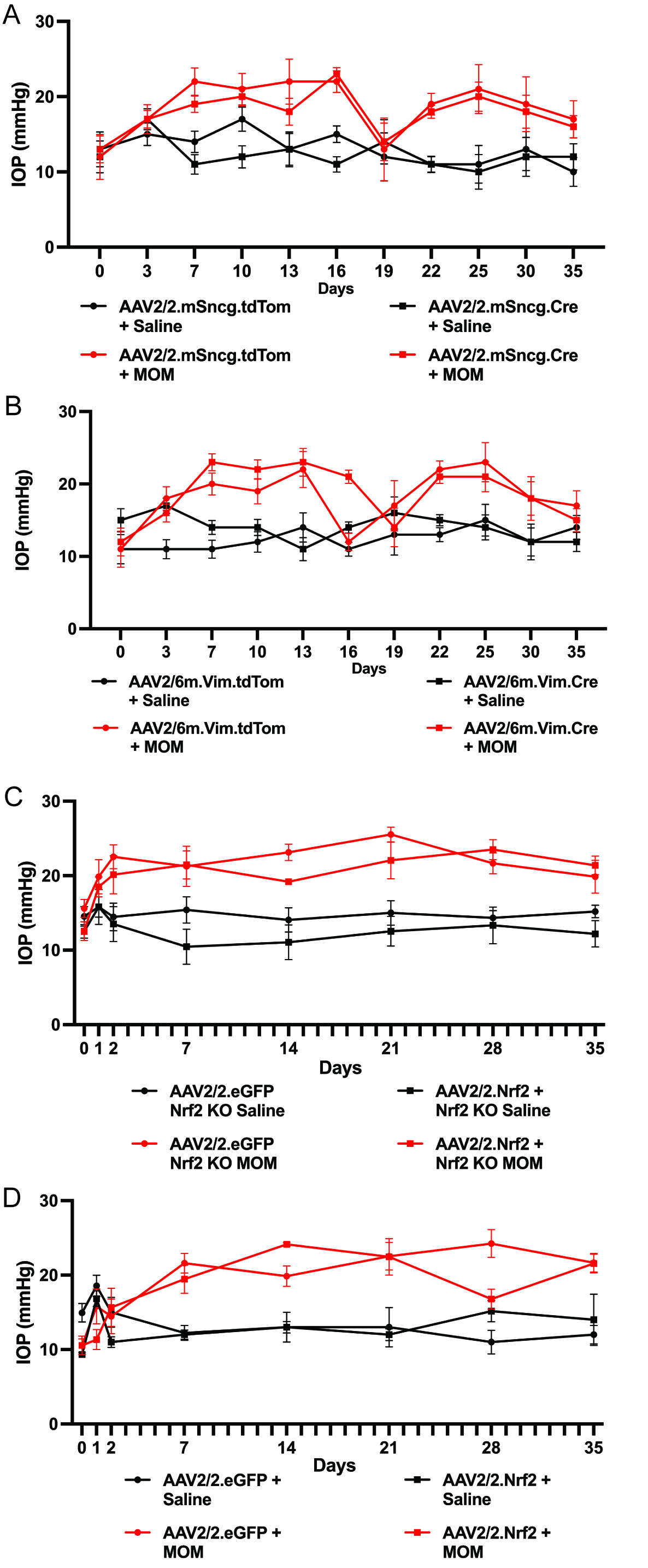

Supplement: Supplementary file 2 — Supplemental Figure 2. IOP was elevated in microbead injected mice. A, B) IOPs from mice used to generate data shown in Figures 1 and 2. Mice were reinjected with microbeads on day 19. C) IOPs from mice used to generate data shown in Figure 4. D) IOPs from mice used to generate data shown in Figure 6. [file 40478_2023_1663_MOESM2_ESM.tif]
